# Vaccine-Elicited Antibodies Restrict Glucose Availability to Control *Brucella* Infection

**DOI:** 10.1093/infdis/jiae172

**Published:** 2024-04-08

**Authors:** Bárbara Ponzilacqua-Silva, Alexis S Dadelahi, Mostafa F N Abushahba, Charles R Moley, Jerod A Skyberg

**Affiliations:** Department of Veterinary Pathobiology, College of Veterinary Medicine; Laboratory for Infectious Disease Research, University of Missouri, Columbia; Department of Veterinary Pathobiology, College of Veterinary Medicine; Laboratory for Infectious Disease Research, University of Missouri, Columbia; Department of Veterinary Pathobiology, College of Veterinary Medicine; Laboratory for Infectious Disease Research, University of Missouri, Columbia; Department of Zoonoses, Faculty of Veterinary Medicine, Assiut University, Egypt; Department of Veterinary Pathobiology, College of Veterinary Medicine; Laboratory for Infectious Disease Research, University of Missouri, Columbia; Department of Veterinary Pathobiology, College of Veterinary Medicine; Laboratory for Infectious Disease Research, University of Missouri, Columbia

**Keywords:** antibody, *Brucella*, brucellosis, metabolism, vaccine

## Abstract

The impact of vaccine-induced immune responses on host metabolite availability has not been well studied. Here we show that prior vaccination alters the metabolic profile of mice challenged with *Brucella melitensis*. In particular, glucose levels were reduced in vaccinated mice in an antibody-dependent manner. We also found the glucose transporter gene *gluP* plays a lesser role in *B melitensis* virulence in vaccinated wild type mice relative to vaccinated mice unable to secrete antibodies. These data indicate that vaccine-elicited antibodies protect the host in part by restricting glucose availability. Moreover, *Brucella* and other pathogens may need to employ different metabolic strategies in vaccinated hosts.

## Teaser

Prior vaccination alters tissue metabolite levels during *Brucella* infection. In particular, vaccine-elicited antibodies lower tissue glucose levels and diminish the role of a glucose transporter in the virulence of *Brucella*.

Brucellosis is a worldwide bacterial zoonosis with a broad host range, including wildlife and agriculturally important livestock [1]. The main species recognized as substantial health threats are *Brucella melitensis*, *B abortus*, and *B suis* [2]. Human brucellosis is a severely debilitating disease characterized by persistent waves of fever that typically requires hospitalization [1]. While live vaccines reduce disease incidence in animals, no vaccine is licensed to prevent human brucellosis [2].

*Brucella* is a facultative intracellular organism that metabolizes a range of carbon sources to promote its growth and/or virulence, including hexoses, pentoses, glycerol, lactate, glutamate, malate, and fatty acids [3–7]. However, it is unknown how vaccine-induced immune responses affect the metabolic conditions that *Brucella* encounters in the host. Here, we performed global screening of host tissue metabolites in *Brucella-*infected mice with and without prior vaccination. In addition, we investigated the effects of host metabolic changes on the requirements for *Brucella* virulence.

## METHODS

### Mice

All animal studies were conducted in compliance with the guidelines of the University of Missouri Animal Care and Use Committee. We utilized 6- to 12-week-old age- and sex-matched animals on a C57BL/6J background. C57BL/6J mice were obtained from Jackson Laboratory, and *sIgM*^–/–^*AID*^–/–^ mice [8, 9] were a gift from Dr Nicole Baumgarth at the University of California, Davis.

### Bacterial Strains and Growth Conditions

*B melitensis* 16M was obtained from Montana State University and *B abortus* S19 from the University of Wyoming. The *gluP* gene (BMEII1053) was previously replaced in frame in *B melitensis* 16M with a chloramphenicol resistance cassette and complemented [10]. The *aceA* gene (BMEI0409) in *B melitensis* 16M was replaced in frame with a chloramphenicol resistance gene in the same manner as described for *gluP* [10] with primers described in Supplementary Table 1. *Brucella* strains were grown on *Brucella* agar (Becton Dickinson) at 37 °C/5% CO_2_. Colonies were transferred to *Brucella* broth (Becton Dickinson) and grown at 37 °C with shaking overnight. *Brucella* concentration was estimated by measuring optical density (OD) at 600 nm, and inoculum was prepared and diluted to the appropriate concentration in sterile phosphate-buffered saline (sPBS). Titer was confirmed by serial dilution of inoculum onto agar.

For vaccination, mice were injected subcutaneously with 2 × 10^5^ colony-forming units (CFUs) of *B abortus* S19 or with sPBS. After 4 weeks of vaccination, animals were challenged intraperitoneally with 1 × 10^5^ CFUs of *B melitensis* 16M. For coinfections, mice were challenged intraperitoneally with an inoculum of 1 × 10^5^ CFUs containing a 1:1 ratio of *B melitensis* 16M and a chloramphenicol-resistant mutant strain (*B melitensis*Δ*gluP* or *B melitensis*Δ*aceA*).

For passive transfer studies, blood was collected via intracardial exsanguination of C57BL/6J mice vaccinated 4 weeks prior with S19 or from naive C57BL/6J mice. Serum samples were centrifuged, pooled, filtered, and stored at −80 °C until transfer. For passive transfer, 200 µL of serum from S19-vaccinated or naive mice was administrated intraperitoneally to recipients 1 day before challenge.

### Calculation of Bacterial Burdens

Spleens were homogenized mechanically in sPBS. Serial dilutions were performed in triplicate in sPBS and plated onto agar. Plates were incubated for 3 days at 37 °C/5% CO_2_, colonies were counted, and CFUs per tissue were calculated. For vaccination experiments, agar was supplemented with 1 mg/mL of erythritol to exclude S19 growth [8]. For competition experiments, bacterial burdens were determined by plating homogenates on agar with or without 5 µg/mL of chloramphenicol. The log_10_ competitive index (log_10_ CI) was calculated by the ratio of bacteria recovery from tissue relative to the ratio in the inoculum [10].

### Metabolite Quantification by Gas Chromatography–Mass Spectrometry

Spleens were weighed, and an approximately 40-mg aliquot was used for gas chromatography–mass spectrometry (GC-MS), with the remainder used to enumerate *Brucella* burdens. GC-MS was performed at the University of Missouri Metabolomics Center as previously described [10]. Briefly, metabolites from homogenized spleens were extracted in 80% methanol, derivatized, and polar and non-polar metabolites were measured by GC-MS. Results were analyzed with MetaboAnalyst 5.0 [11] with a fold change cutoff of 1.5 and an adjusted *P* value threshold of .05.

### RNA Sequencing

Spleens were homogenized in Trizol Reagent (Thermo Fisher) and RNA isolated according to the manufacturer's instructions. RNA was further purified with RNeasy columns (Qiagen). Poly(A)-enriched stranded mRNA libraries were generated, which were sequenced on a NovaSeq 6000 (Illumina). RNA sequencing analysis was performed with OneStopRNAseq [8, 12]. These data were deposited into the NCBI Gene Expression Omnibus and are accessible through accession GSE254248.

### Glucose Quantification in Spleens

Spleen homogenates were frozen at −80 °C, thawed, and centrifuged, and glucose levels in supernatants were quantified with the Glucose Assay Kit II (Eton Biosciences) according to the manufacturer's instructions.

### Effect of Lyxonate and Glucose on *Brucella* Growth In Vitro

Overnight cultures of *Brucella* were centrifuged and washed with sPBS 3 times. The OD at 600 nm was measured, and cultures were resuspended at an OD of ∼0.05 in Luria-Bertani broth (Becton Dickinson) with or without 0.4% glucose or 0.4% lyxonate (Carbosynth). Samples were then cultured in a 96-well plate and incubated at 37 °C/5% CO_2_, and OD measurements were made 24 and 48 hours later.

### Statistical Analysis

All data are expressed as mean and SD. A Student unpaired *t* test was used to assess differences in means between 2 groups, while analysis of variance, followed by a Tukey test, was used for comparisons among ≥3 groups. Significance was set at *P* < .05

Simple linear regression was used to model the relationship between 2 continuous variables. All statistical analyses were performed with Prism software (version 10.1.1; GraphPad).

## RESULTS

### Vaccination Prior to Challenge Alters the Metabolic and Transcriptomic Profile of Tissues Infected With *Brucella*

**Figure 1.**
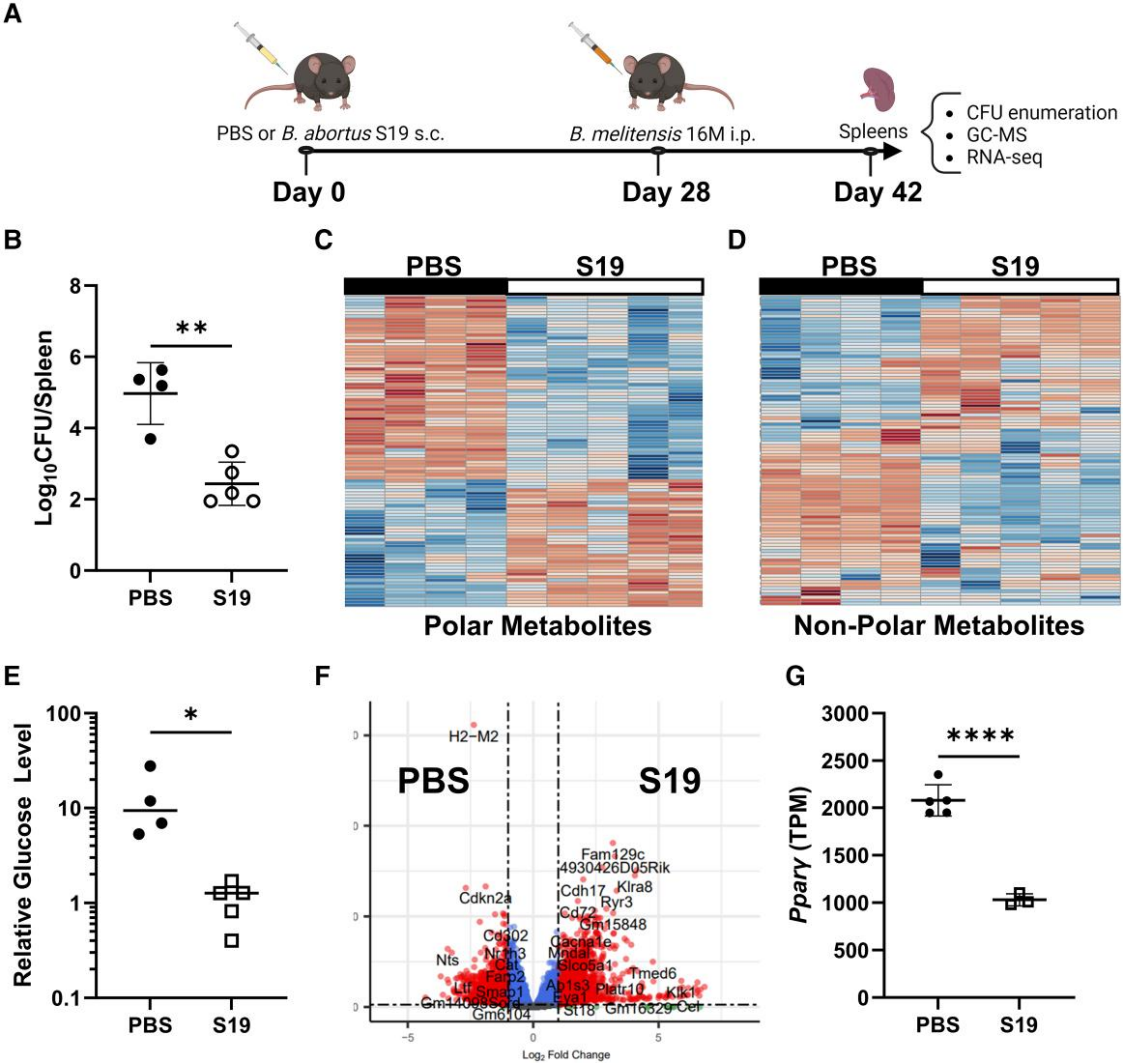
Prior vaccination alters the metabolic and transcriptomic profile of spleens in mice challenged with *Brucella melitensis*. *A*, General experimental design for Figure 1 (image created with Biorender.com). *B–E*, C57BL/6J mice (n = 4 or 5/group) were vaccinated subcutaneously with 2 × 10^5^ CFUs of S19 or treated with PBS 4 weeks prior to intraperitoneal challenge with 1 × 10^5^ CFUs of *B melitensis* 16M. Two weeks after challenge, splenic levels of *B melitensis* were determined (*B*), and GC-MS was performed to generate a heat map of polar (*C*) and nonpolar (*D*) metabolites in the spleens of mice that received PBS vs S19. *E*, Relative glucose levels in spleens as determined by GC-MS 2 weeks after challenge. *F* and *G*, Wild type mice (n = 3–5/group) were treated with PBS or vaccinated subcutaneously with S19 4 weeks prior to intraperitoneal challenge with *B melitensis* 16M (1 × 10^5^ CFUs). Two weeks postchallenge, RNA sequencing was performed on RNA isolated from spleens. *F*, Volcano plot demonstrating downregulated (left) and upregulated (right) gene expression between groups. The horizontal dashed lines indicate a false discovery rate of 0.05, and genes outside the vertical dashed lines have a 2-fold absolute change. *G*, Transcripts per million (TPM) of *Pparg* in S19-vaccinated and PBS-treated mice 2 weeks after *B melitensis* infection. Data are representative from 1 experiment and presented as mean (SD). *P < .05. **P < .01. ****P < .0001. *t* test. CFU, colony-forming unit; GC-MS, gas chromatography–mass spectrometry; ip, intraperitoneally; PBS, phosphate-buffered saline; sc, subcutaneously.

*B abortus* strain S19 (S19) is a smooth live-attenuated vaccine commonly used to prevent brucellosis in cattle that is cross-protective against *B melitensis* [8]. We vaccinated wild type (WT) C57BL/6J mice with S19 or treated them with phosphate-buffered saline (PBS) as a control 4 weeks prior to challenge with *B melitensis*. After 2 weeks of challenge, spleens were harvested for measuring CFUs, metabolomics, and RNA sequencing (Figure 1*A*). S19 vaccination conferred approximately 100-fold protection against *B melitensis* (Figure 1*B*). In addition, GC-MS revealed differences in metabolite availability in mice vaccinated prior to challenge (Figure 1*C* and 1*D*). A total of 341 polar or nonpolar metabolites were identified, 206 of which were altered at least 1.5-fold by vaccination (Supplementary Tables 2 and 3).

When investigating individual metabolites, we found that lyxonate levels were increased approximately 3-fold while glucose levels were decreased approximately 10-fold in spleens of mice vaccinated prior to challenge (Supplementary Figure 1A and Figure 1*E*). While lyxonate can serve as a carbon source for *Pseudomonas aeruginosa* [13], we noted that lyxonate modestly inhibited *B melitensis* growth in vitro (Supplementary Figure 1B and 1C). *Brucella* employs the glucose transporter *gluP* to promote growth in broth and virulence in vivo [10, 14, 15]. In vitro, *Brucella* utilizes GluP to replicate in proliferator-activated receptor γ, expressing alternatively activated macrophages due to elevated glucose in these cells [14]. Interestingly, RNA sequencing revealed that *Pparg* expression was diminished in the spleens of *Brucella*-infected mice that were vaccinated prior to challenge (Figure 1*F* and 1*G* and Supplementary Table 4). Levels of eicosapentaenoic acid, which promotes alternative macrophage activation [16], were also lower in vaccinated mice (Supplementary Figure 1D). Therefore, we became interested in whether vaccination alters host glucose availability to control infection.

### Vaccine-Elicited Antibodies Restrict Tissue Glucose Availability and Diminish the Role for *Glup* in *B Melitensis* Virulence

**Figure 2.**
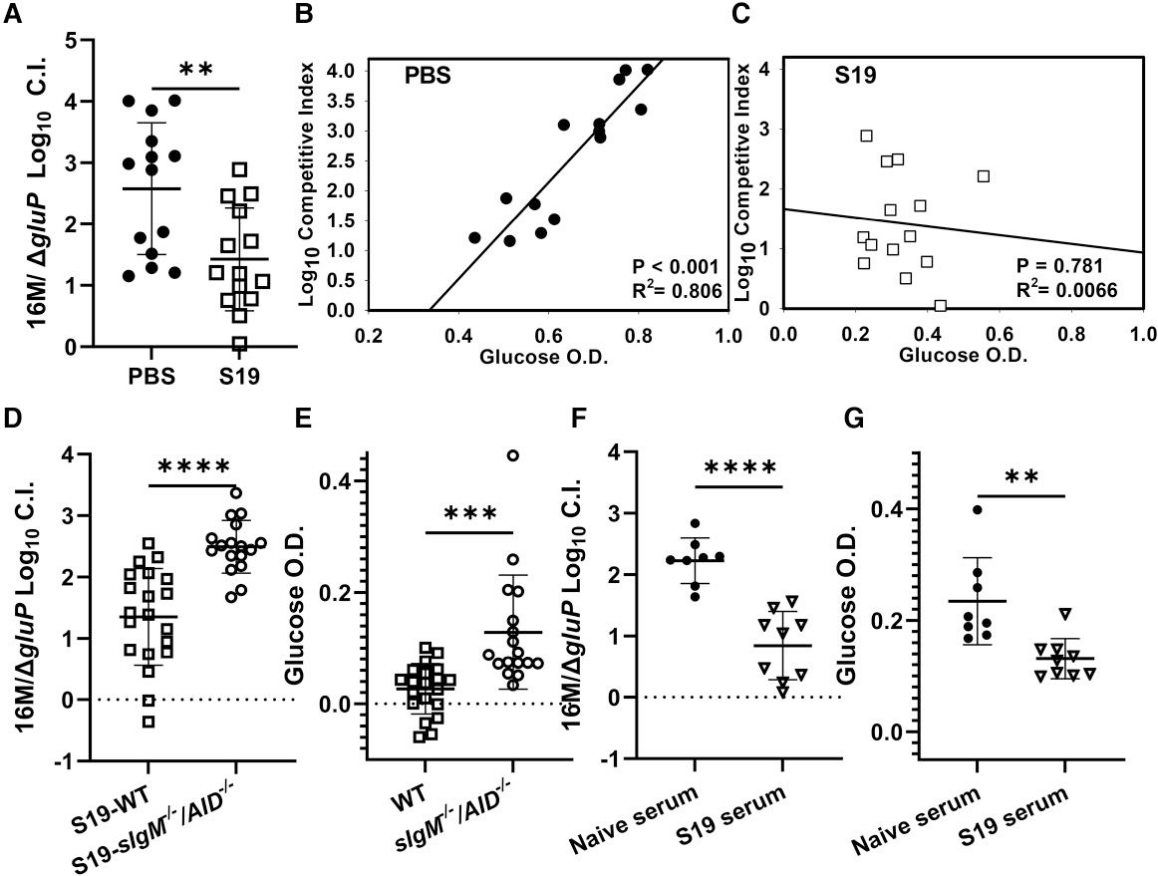
Vaccine-elicited antibodies restrict glucose availability to control *Brucella melitensis* infection. *A–B*, C57BL/6J (WT) mice (n = 14/group) were treated with PBS or vaccinated subcutaneously with S19 (2 × 10^5^ CFUs) 4 weeks prior to challenge with 1 × 10^5^ CFUs of a 1:1 mix of WT *B melitensis* 16M and *B melitensis*Δ*gluP*. *A*, Two weeks postinfection, a log_10_ competitive index (CI) based on relative strain recovery was calculated. *B* and *C*, Glucose levels in spleens were measured via colorimetric assay and plotted against the log_10_ CI to perform linear regressions. WT or *sIgM^−/−^AID^−/−^* mice (n = 17–20/group) were vaccinated subcutaneously with S19 (2 × 10^5^ CFUs) 4 weeks prior to intraperitoneal challenge with 1 × 10^5^ CFUs of a 1:1 mix of WT *B melitensis* 16M and *B melitensis*Δ*gluP*. Two weeks postinfection, a log_10_ CI based on relative strain recovery was calculated (*D*), and glucose levels in spleens were measured via colorimetric assay (*E*). *F* and *G*, Sterile filtered serum from WT naïve mice (200 µL) or from WT mice vaccinated subcutaneously 4 weeks prior with 2 × 10^5^ CFUs of S19 was transferred intraperitoneally to naive WT recipients (n = 8 or 9/group) 1 day prior to intraperitoneal challenge with 1 × 10^5^ CFUs of a 1:1 mix of 16M and *Bm*Δ*gluP*. Two weeks postinfection, a log_10_ CI was calculated (*F*), and glucose levels in spleens were measured (*G*). Data are combined from 2 or 3 experiments and presented as mean (SD). **P < .01. ***P < .001. ****P < .0001. *t* test. *B* and *C*, *P* values were determined via linear regression. CFU, colony-forming unit; OD, optical density; PBS, phosphate-buffered saline; WT, wild type.

To determine if glucose restriction plays a role in vaccine-mediated immunity, we treated mice with PBS or vaccinated them with S19 4 weeks prior to challenge with a 1:1 ratio of WT *B melitensis* and a chloramphenicol-resistant mutant lacking *gluP*, *B melitensis*Δ*gluP*. Two weeks after challenge, we determined the ratio of WT *B melitensis* to *B melitensis*Δ*gluP* in spleens by a log_10_ CI based on relative strain recovery, where 0 indicates that the mutant is not attenuated and 1 indicates that the mutant is attenuated 10-fold [10]. Remarkably, the log_10_ CI was >10-fold lower in vaccinated animals as compared with PBS-treated mice, indicating that vaccination diminishes the role for *gluP* in *Brucella* virulence (Figure 2*A*). We also found that glucose levels were positively correlated with attenuation of *B melitensis*Δ*gluP* in animals treated with PBS prior to challenge (Figure 2*B*) but not in vaccinated animals (Figure 2*C*). Together these data demonstrate that S19 vaccination protects the host in part by restricting tissue glucose availability.

Studies with B cell–deficient mice have indicated that humoral immunity contributes to protection during secondary infection with *B melitensis* [17]. In addition, by using *sIgM^−/−^AID^−/−^* mice unable to secrete IgM or class-switched antibodies, we determined that S19-mediated immunity against *B melitensis* requires antibody production [8]. To explore the potential connection between antibody production and glucose restriction, we vaccinated WT and *sIgM^−/−^AID^−/−^* mice with S19 4 weeks prior to coinfection with WT *B melitensis* and *B melitensis*Δ*gluP*. Two weeks postchallenge, we noted that the log_10_ CI was approximately 10-fold higher in *sIgM^−/−^AID^−/−^* mice as compared with WT animals (Figure 2*D*), indicating that *B melitensis*Δ*gluP* is more highly attenuated in vaccinated animals unable to produce antibodies relative to vaccinated WT mice. We also found that glucose levels were significantly higher in vaccinated *sIgM^−/−^AID^−/−^* animals as compared with vaccinated WT mice (Figure 2*E*). This effect appears to be specific for vaccination, as relative glucose levels and the attenuation of *B melitensis*Δ*gluP* did not vary in unvaccinated WT vs *sIgM^−/−^AID^−/−^* mice (Supplementary Figure 2A and 2B).

To confirm that the observed phenotype was conferred by antibodies and not cell-mediated immunity, we performed passive transfer experiments using serum from naive animals or mice vaccinated with S19. Serum was transferred to WT mice a day before coinfection with WT *B melitensis* and *B melitensis*Δ*gluP.* S19 serum transfer resulted in significantly lower relative attenuation of *B melitensis*Δ*gluP* as compared with naive serum (Figure 2*F*). Corroborating this result, immune serum transfer reduced splenic glucose levels (Figure 2*G*). These data collectively indicate that vaccination reduces relative glucose availability in spleens and diminishes the role of *gluP* in *Brucella* virulence in an antibody-dependent manner.

## DISCUSSION

*Brucella* is highly adaptable inside hosts and can use a variety of carbon sources for growth [5]. Here we investigated how induction of host immune responses by vaccination alters metabolite availability and the metabolic requirements for *Brucella* virulence. We determined that the metabolic and transcriptomic profile of animals vaccinated prior to challenge was distinct from PBS-treated animals (Figure 1). Interestingly, by employing *sIgM^−/−^AID^−/−^* mice unable to secrete antibodies and by passive transfer of immune serum, we show that vaccine-elicited antibodies lower tissue glucose levels and diminish the role for *gluP* in *B melitensis* virulence (Figure 2). These findings could be due to altered host cell activation, as antibodies can engage Fc receptors or activate complement, which can trigger host cell glycolysis and therefore restrict glucose availability [18, 19]. In these conditions, lower glucose levels would diminish growth of WT *B melitensis* but not *B melitensis*Δ*gluP*, which matches our results where we found that the ratio of WT *B melitensis* to *B melitensis*Δ*gluP* is higher in the absence of vaccine-elicited antibodies (Figure 2).

Restriction of glucose availability by vaccine-elicited antibodies likely causes *Brucella* to rely on carbon sources other than glucose to sustain growth and/or virulence. When glycolytic substrates become scarce, *Mycobacterium tuberculosis* employs isocitrate lyases to β-oxidate fatty acids to provide energy [20]. *B melitensis* does encode an isocitrate lyase called *aceA*. However, *aceA* has been reported to be dispensable for *Brucella* virulence [3, 4], and we did not find *aceA* to play a differential role in virulence in vaccinated vs naive hosts (Supplementary Figure 2C). Therefore, if *B melitensis* does β-oxidate fatty acids to sustain virulence in vaccinated hosts, it likely employs isocitrate lyase–independent mechanisms, such as *fadA* and *fadJ*, which are implicated in fatty acid β-oxidation and required for *B melitensis* virulence [7]. Alternatively, in a vaccinated host, *Brucella* could use carbon sources other than sugars or fatty acids, as it known that genes associated with metabolism of lactate, glycerol, and glutamate are required for *Brucella* virulence [5, 6, 15, 21].

Collectively, our data indicate that vaccine-elicited antibodies reduce glucose availability to protect the host against *Brucella*. In addition, *Brucella* and other pathogens may need to employ different metabolic virulence strategies in vaccinated vs naive hosts.

## Supplementary Data

Supplementary materials are available at *The Journal of Infectious Diseases* online (http://jid.oxfordjournals.org/). Supplementary materials consist of data provided by the author that are published to benefit the reader. The posted materials are not copyedited. The contents of all supplementary data are the sole responsibility of the authors. Questions or messages regarding errors should be addressed to the author.

## Supplementary Material

jiae172_Supplementary_Data

## Notes

***Financial support***. This work was supported by the National Institutes of Health (grants R01AI150797 and R21AI146397 to J. A. S.); and funding from the University of Missouri Metabolomics Center.

## References

[1] European Food Safety Authority and European Centre for Disease Prevention and Control . The European Union one health 2019 zoonoses report. EFSA J 2021; 19:e06406. 33680134 10.2903/j.efsa.2021.6406 PMC7913300

[2] Corbel MJ . Brucellosis: an overview. Emerg Infect Dis 1997; 3:213–21. 9204307 10.3201/eid0302.970219 PMC2627605

[3] Kohler S , Foulongne V, Ouahrani-Bettache S, et al The analysis of the intramacrophagic virulome of *Brucella suis* deciphers the environment encountered by the pathogen inside the macrophage host cell. Proc Natl Acad Sci U S A 2002; 99:15711–6. 12438693 10.1073/pnas.232454299 PMC137781

[4] Zuniga-Ripa A , Barbier T, Conde-Alvarez R, et al *Brucella abortus* depends on pyruvate phosphate dikinase and malic enzyme but not on Fbp and GlpX fructose-1,6-bisphosphatases for full virulence in laboratory models. J Bacteriol 2014; 196:3045–57. 24936050 10.1128/JB.01663-14 PMC4135635

[5] Barbier T , Zuniga-Ripa A, Moussa S, et al *Brucella* central carbon metabolism: an update. Crit Rev Microbiol 2018; 44:182–211. 28604247 10.1080/1040841X.2017.1332002

[6] Czyz DM , Willett JW, Crosson S. *Brucella abortus* induces a Warburg shift in host metabolism that is linked to enhanced intracellular survival of the pathogen. J Bacteriol 2017; 199:e00227-17. 28559292 10.1128/JB.00227-17 PMC5512224

[7] Potemberg G , Demars A, Barbieux E, et al Genome-wide analysis of *Brucella melitensis* genes required throughout intranasal infection in mice. PLoS Pathog 2022; 18:e1010621. 35771771 10.1371/journal.ppat.1010621 PMC9246152

[8] Abushahba MFN , Dadelahi AS, Ponzilacqua-Silva B, Moley CR, Skyberg JA. Contrasting roles for IgM and B cell MHCII expression in *Brucella abortus* S19 vaccine-mediated efficacy against *B melitensis* infection. mSphere 2024; 9:e00750-23. 38349167 10.1128/msphere.00750-23 PMC10964430

[9] Dadelahi AS , Abushahba MFN, Ponzilacqua-Silva B, et al Interactions between B cells and T follicular regulatory cells enhance susceptibility to *Brucella* infection independent of the anti-*Brucella* humoral response. PLoS Pathog 2023; 19:e1011672. 37721965 10.1371/journal.ppat.1011672 PMC10538787

[10] Lacey CA , Ponzilacqua-Silva B, Chambers CA, Dadelahi AS, Skyberg JA. MyD88-dependent glucose restriction and itaconate production control *Brucella* infection. Infect Immun 2021; 89:e0015621. 34125603 10.1128/IAI.00156-21 PMC8445166

[11] Xia J , Psychogios N, Young N, Wishart DS. MetaboAnalyst: a web server for metabolomic data analysis and interpretation. Nucleic Acids Res 2009; 37:W652–60. 19429898 10.1093/nar/gkp356 PMC2703878

[12] Li R , Hu K, Liu H, Green MR, Zhu LJ. OneStopRNAseq: a web application for comprehensive and efficient analyses of RNA-Seq data. Genes (Basel) 2020; 11:1165. 33023248 10.3390/genes11101165 PMC7650687

[13] Ghasempur S , Eswaramoorthy S, Hillerich BS, et al Discovery of a novel L-lyxonate degradation pathway in *Pseudomonas aeruginosa* PAO1. Biochemistry 2014; 53:3357–66. 24831290 10.1021/bi5004298 PMC4038344

[14] Xavier MN , Winter MG, Spees AM, et al PPARgamma-mediated increase in glucose availability sustains chronic *Brucella abortus* infection in alternatively activated macrophages. Cell Host Microbe 2013; 14:159–70. 23954155 10.1016/j.chom.2013.07.009 PMC3777723

[15] Hong PC , Tsolis RM, Ficht TA. Identification of genes required for chronic persistence of *Brucella abortus* in mice. Infect Immun 2000; 68:4102–7. 10858227 10.1128/iai.68.7.4102-4107.2000 PMC101704

[16] Videla LA , Valenzuela R, Del Campo A, Zuniga-Hernandez J. Omega-3 lipid mediators: modulation of the M1/M2 macrophage phenotype and its protective role in chronic liver diseases. Int J Mol Sci 2023; 24:15528. 37958514 10.3390/ijms242115528 PMC10647594

[17] Vitry MA , Hanot Mambres D, De Trez C, et al Humoral immunity and CD4+ Th1 cells are both necessary for a fully protective immune response upon secondary infection with *Brucella melitensis*. J Immunol 2014; 192:3740–52. 24646742 10.4049/jimmunol.1302561

[18] Jing C , Castro-Dopico T, Richoz N , et al. Macrophage metabolic reprogramming presents a therapeutic target in lupus nephritis. Proc Natl Acad Sci U S A 2020; 117:15160–71. 32541026 10.1073/pnas.2000943117 PMC7334513

[19] Hess C , Kemper C. Complement-mediated regulation of metabolism and basic cellular processes. Immunity 2016; 45:240–54. 27533012 10.1016/j.immuni.2016.08.003 PMC5019180

[20] Munoz-Elias EJ , McKinney JD. *Mycobacterium tuberculosis* isocitrate lyases 1 and 2 are jointly required for in vivo growth and virulence. Nat Med 2005; 11:638–44. 15895072 10.1038/nm1252 PMC1464426

[21] Lestrate P , Dricot A, Delrue RM, et al Attenuated signature-tagged mutagenesis mutants of *Brucella melitensis* identified during the acute phase of infection in mice. Infect Immun 2003; 71:7053–60. 14638795 10.1128/IAI.71.12.7053-7060.2003 PMC308902

