# Supplementary material for: Vaccine-Elicited Antibodies Restrict Glucose Availability to Control Brucella Infection
Source: J Infect Dis. 2024 Apr 8;230(4):e818–23. doi: 10.1093/infdis/jiae172 (PMC11481323; doi:10.1093/infdis/jiae172)
Supplement: jiae172_Supplementary_Data [file jiae172_supplementary_data.zip › SupplementalFigure1.docx]

**Supplemental Figure 1.**


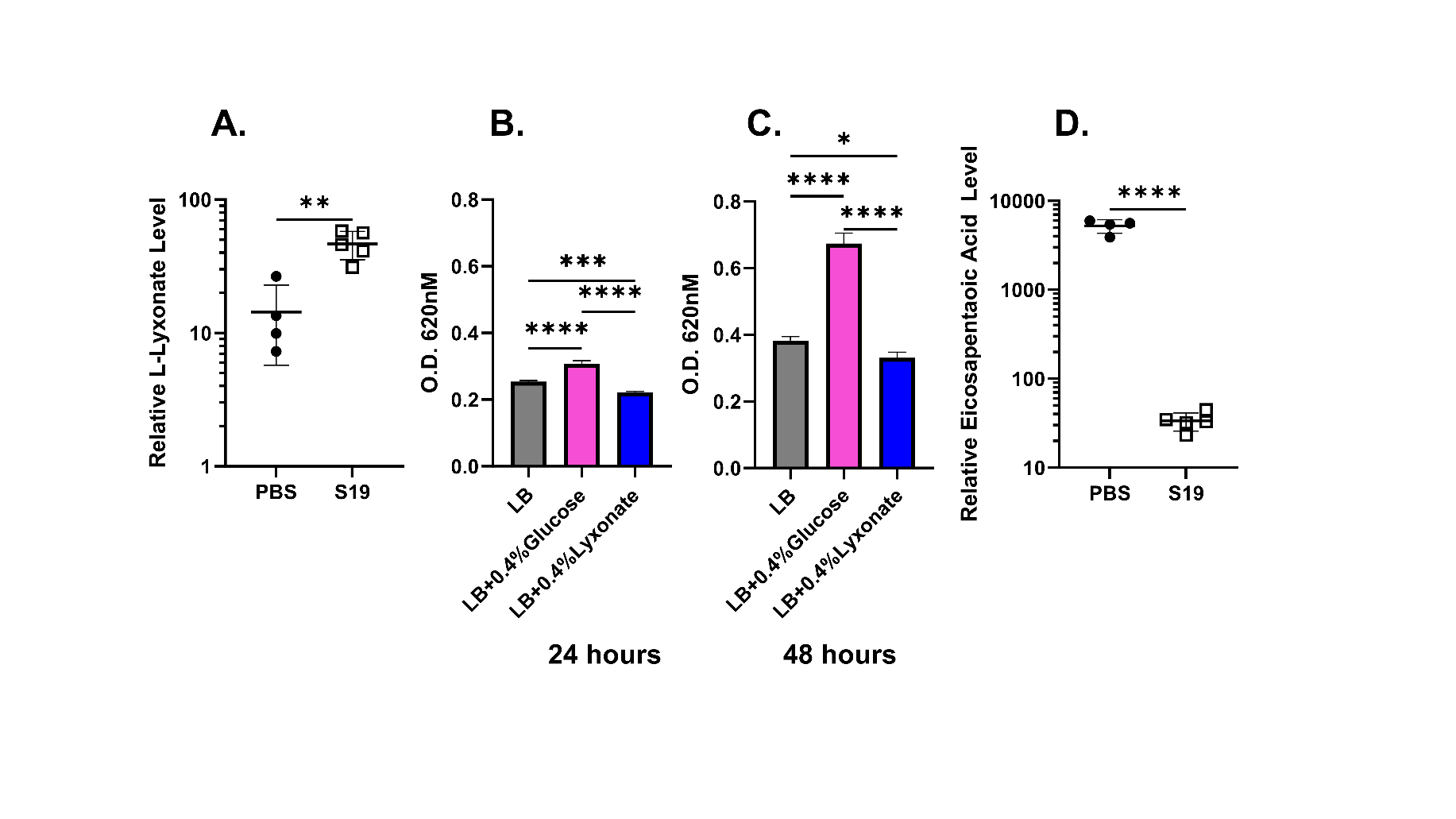


**Figure S1.: Glucose, but not lyxonate, promotes *B. melitensis* growth. A,D)** C57BL/6J mice (n=4-5/group) were vaccinated s.c with 2x10^5^ CFUs of S19 or treated with PBS four weeks prior to i.p. challenge with 1x10^5^ CFUs of *B. melitensis* 16M. GC-MS was performed two weeks after challenge to determine relative levels of lyxonate **A)** and eicosapentaenoic acid **D)** in spleens. **B-C)** *B. melitensis* 16M was grown in LB broth alone, or LB broth supplemented with 0.4% glucose or 0.4% lyxonate. O.D. measurements were made after 24 **B)** and 48 **C)** hours of culture. Data in **A,D)** is representative of one experiment while data in **B-C)** is representative of two experiments. *P<0.05, ** P<0.01, *** P<0.001, **** P<0.0001 via T-test **A,D)** or ANOVA with Tukey’s post-hoc test **B-C)**.
