# Supplementary material for: Vaccine-Elicited Antibodies Restrict Glucose Availability to Control Brucella Infection
Source: J Infect Dis. 2024 Apr 8;230(4):e818–23. doi: 10.1093/infdis/jiae172 (PMC11481323; doi:10.1093/infdis/jiae172)
Supplement: jiae172_Supplementary_Data [file jiae172_supplementary_data.zip › SupplementalFigure2.docx]

**Supplemental Figure 2**

**
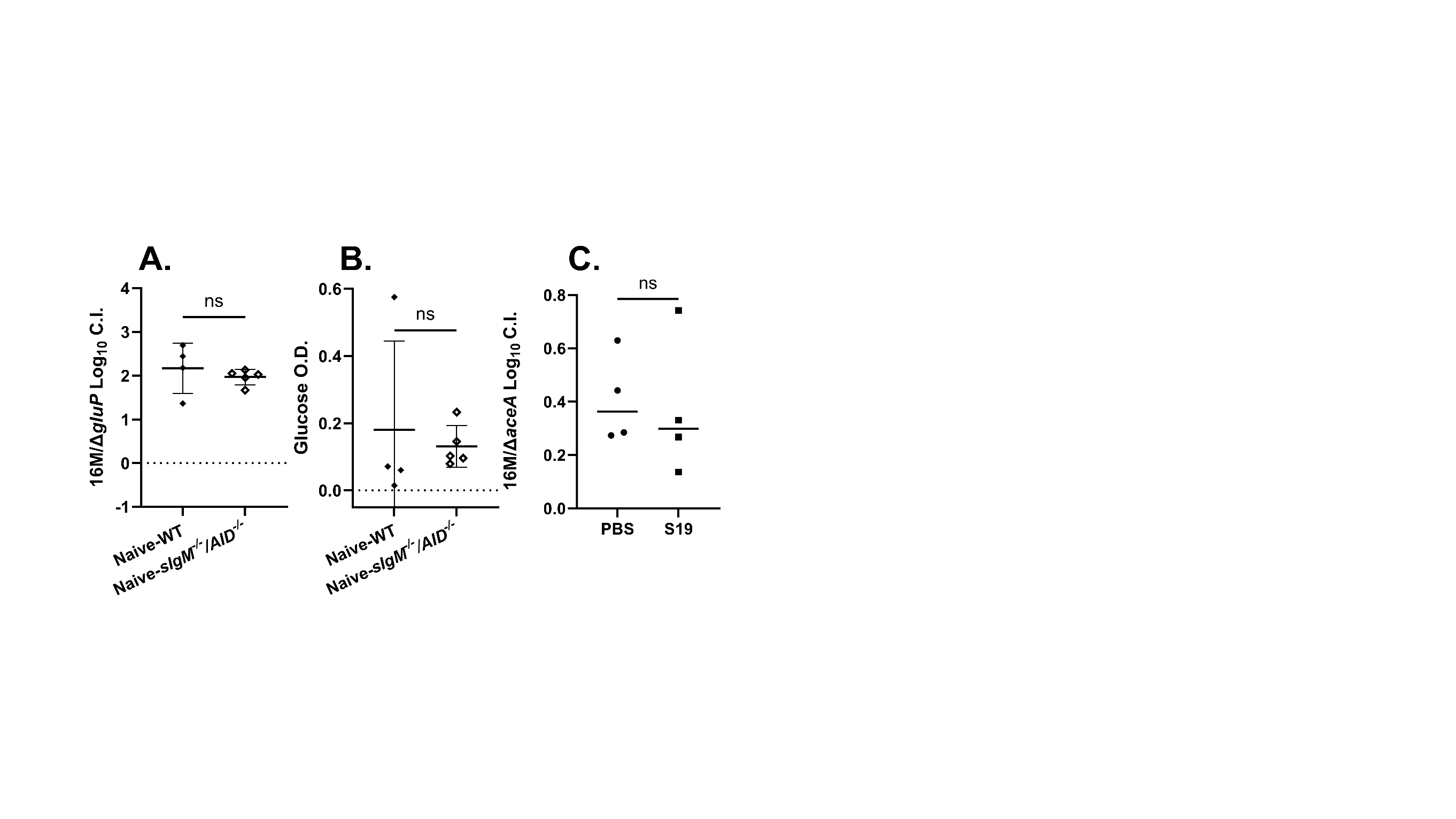
**

**Figure S2. aceA does not differentially contribute to B. melitensis virulence in a vaccinated host.** Naïve C57BL/6J mice (WT) or sIgM^-/-^/AID^-/-^ mice (n=4-5/group) were challenged i.p. with 1x10^5^ CFUs of a 1:1 mix of WT B. melitensis 16M “16M” and B. melitensisΔgluP. Two weeks post-infection a Log_10_ C.I. based on relative strain recovery was calculated **A)** and glucose levels in spleens were measured via colorimetric assay **B)**. WT mice (n=4/group) were treated with PBS or vaccinated s.c. with S19 (2x10^5^ CFUs) four weeks prior to challenge with 1x10^5^ CFUs of a 1:1 mix of WT B. melitensis 16M “16M” and B. melitensisΔaceA. Two weeks post-infection, a Log_10_ Competitive Index (C.I.) based on relative strain recovery was calculated **C).** Data are from one experiment. ns= not significant via T-test.
