# Supplementary material for: Vaccine-Elicited Antibodies Restrict Glucose Availability to Control Brucella Infection
Source: J Infect Dis. 2024 Apr 8;230(4):e818–23. doi: 10.1093/infdis/jiae172 (PMC11481323; doi:10.1093/infdis/jiae172)
Supplement: jiae172_Supplementary_Data [file jiae172_supplementary_data.zip › TableS1.docx]

**Supplementary Table 1**.

Primers used in this study.

| Primer name | 5’-3’ sequence | Purpose |
| --- | --- | --- |
| aceA-up-F* | GCGCCAGAAAGCTTCCTGCAGGATATCGTGTGAGCCCTTCCGTTTCCACACCG | Cloning fragment upstream of *aceA* |
| aceA-up-R | ATCTGTCATTTCGGTGTCTCCTCG | Cloning fragment upstream of *aceA* |
| aceA-cat-F^ | TCACCACGAGGAGACACCGAAATGACAGATGTGTAGGCTGGAGCTGCTTC | Cloning chloramphenicol resistance gene |
| aceA-cat-R | CATATGAATATCCTCCTTA | Cloning chloramphenicol resistance gene |
| aceA-downstream F^#^ | GGAATAGGAACTAAGGAGGATATTCATATGGCCGAGTAGCAGCCGGGACCACCG | Cloning fragment downstream of *aceA* |
| gluP downstream R* | CCAAGCTACGTAATACGACTCACTAGTGGGAAGGCAGCATGTGTCATGCACAGG | Cloning fragment downstream of *aceA* |
| aceA seq F | GATATCACCACGAGGAGACACCG | Screening of *aceA* mutant |
| aceA seq R | TCCTGGTTCGGTGGTCCCGGCTGC | Screening of *aceA* mutant |

*Underlined regions denote homology to pBBR1MCS-2

^Underlined region denote homology to *aceA* upstream fragment

^#^Underlined region denote homology to cloned chloramphenicol resistance gene

All primers were from IDT, Coralville, IA
